# Multifunctional lignin-poly (lactic acid) biocomposites for packaging applications

**DOI:** 10.3389/fbioe.2022.1025076

**Published:** 2022-10-03

**Authors:** Esakkiammal Sudha Esakkimuthu, David DeVallance, Ievgen Pylypchuk, Adrian Moreno, Mika H. Sipponen

**Affiliations:** ^1^ InnoRenew CoE, Izola, Slovenia; ^2^ Faculty of Mathematics, Natural Sciences, and Information Technologies, University of Primorska, Koper, Slovenia; ^3^ Department of Materials and Environmental Chemistry, Stockholm University, Stockholm, Sweden

**Keywords:** lignin, polylactic acid, modification, packaging, polymer, matrix, composites

## Abstract

Lignin is the most abundant aromatic biopolymer with many promising features but also shortcomings as a filler in polymer blends. The main objective of this work was to improve the processability and compatibility of lignin with poly (lactic acid) (PLA) through etherification of lignin. Commercial kraft lignin (KL) and oxypropylated kraft lignin (OPKL) were blended with PLA at different weight percentages (1, 5, 10, 20, and 40%) followed by injection molding. Low lignin contents between 1 and 10% generally had a favorable impact on mechanical strength and moduli as well as functional properties of the PLA-based composites. Unmodified lignin with free phenolic hydroxyl groups rendered the composites with antioxidant activity, as measured by radical scavenging and lipid peroxidation tests. Incorporating 5–10% of KL or OPKL improved the thermal stability of the composites within the 300–350°C region. DSC analysis showed that the glass transition temperature values were systematically decreased upon addition of KL and OPKL into PLA polymer. However, low lignin contents of 1 and 5% decreased the cold crystallization temperature of PLA. The composites of KL and OPKL with PLA exhibited good stabilities in the migration test, with values of 17 mg kg^−1^ and 23 mg kg^−1^ even at higher lignin content 40%, i.e., well below the limit defined in a European standard (60 mg kg^−1^). These results suggest oxypropylated lignin as a functional filler in PLA for safe and functional food packaging and antioxidant applications.

## Introduction

Depletion of fossil-based energy resources and increasing environmental pollution are serious concerns that require phasing out of fossil-based plastics with bio-based and biodegradable polymer materials. Among the bio-based polymers available in the market, poly (lactic acid) (PLA) is a commercially available polyester derived from plant materials such as corn starch (Li et al., 2003). While it shows good melt processing properties and ductility, for food packaging applications, PLA’s high brittleness, slow crystallization, poor gas and water vapor barrier properties, and low mechanical and thermal properties restrict its broader utilization (Fortunati et al., 2010; Yang et al., 2015; Murariu and Dubois, 2016). Also, these limitations severely limit the use of PLA in long self-life storage applications. To enhance PLA’s properties without compromising its sustainability, a variety of natural polymers such as cellulose, starch, and lignin have been introduced into the PLA polymer matrix (Saito et al., 2012; Frone et al., 2013; Hassan et al., 2019; Nazrin et al., 2020).

Among natural polymer fillers lignin is a particularly interesting macromolecule owing to its abundant availability and unique properties compared to polysaccharides. It is a three-dimensional aromatic biopolymer, comprised of three basic monomeric units, p-hydroxyphenyl, syringyl, and guaiacyl, interlinked *via* carbon-carbon and ether linkages originating from radical polymerization reactions. Lignin is predominantly obtained as a byproduct from the pulp and paper industry during the pulping process, in which 98% is incinerated for internal energy recovery and only 2% is isolated for commercial purposes (Schoenherr et al., 2017). Around 1.5–1.8 million tons of isolated lignin is used annually (Cao et al., 2018). The aromatic structure, broad availability, and variety of functional groups (-OH, -COOH and -OCH_3_) make lignin suitable for many potential applications, especially polymeric materials (Duval and Lawoko, 2014; Upton and Kasko, 2016; Wang et al., 2016).

Despite some of lignin’s advantageous properties, it is known that the amphiphilic nature of lignin poses challenges to achieving high-quality polymer blends. Lignin has been reported to form aggregates in the polymeric matrix due to pi-pi stacking of aromatic rings, hydrogen bonding between the hydroxyl groups and van der Waals attraction among the polymer chains, which finally results in the poor composite properties (Sarkanen et al., 1981; Glasser and Sarkanen, 1989; Sun et al., 2015; Vaidya et al., 2019; Hong and Hwang, 2021). Chemical modification such as acetylation of the lignin hydroxyl groups has been performed to improve its compatibility in a polymer matrix (Kim et al., 2017). A Comparison of extracted kraft lignin and acetylated kraft lignin in PLA-based composites showed an improved thermal degradation temperature and hydrophobicity with acetylated lignin, but the incorporation of more than 5 wt% of lignin caused a drastic loss in mechanical strength (Gordobil et al., 2015). PLA-lignin composites containing hardwood and softwood-derived lignin showed comparable mechanical strength to pure PLA at lignin contents up to 7% and no significant changes after accelerated weathering trials. However, the water sorption capacity was substantially increased for lignin-PLA composites, and the incorporated lignin additionally provided excellent UV barrier properties (Spiridon et al., 2015). Other research indicated that organosolv lignin, due to its low molecular weight and lower dispersity exhibited higher compatibility with PLA compared to kraft lignin or lignosulfonate when extruded and three-dimensionally printed (Mimini et al., 2019).

On the other hand, a recent study was mainly focused on enhancing the compatibility and processability between lignin and PLA by performing copolymerization with bioderived monomers lauryl methacrylate and tetrahydrofurfuryl methacrylate. The functionalized lignin exhibited a uniform distribution with PLA that led to increased ductility and toughness, and the co-grafted lignin dissipated the fracture energy during the tension process (Sun et al., 2020). Furthermore, the simulated body fluid and *in vitro* studies were conducted for lignin-PLA composites in order to evaluate the degradation of the matrix under physiological conditions along with biocompatibility and toxicity aspects. The addition of lignin did not change the biodegradability or metabolic activity under the physiological conditions in the presence of PLA (Spiridon and Tanase, 2018). These studies show that incorporating lignin in PLA can give new functionalities without compromising mechanical performance. However, many key properties of PLA-lignin composites remain unknown when it comes to material requirements for food applications.

The present work was conducted to improve PLA/Lignin compatibility in view of possible use as food-grade packaging material. PLA-lignin composites with different weight percentages of oxypropylated lignin were produced through a combined blending and injection molding process. The mechanical (tensile strength, Young’s modulus), thermal (glass transition temperature) and chemical properties were examined for lignin/PLA and modified lignin/PLA composites. In addition, the produced specimens were subjected to antioxidant, lipid peroxidation, migration, and biodegradation tests. The results give a comprehensive view of the possibilities and limitations of lignin as a functional filler in PLA composites intended for food packaging specific to container applications.

## Materials and methods

### Materials

Kraft softwood lignin (UPM Biopiva 395) was purchased from UPM biochemicals with an average molecular weight of 6,000 g/mol and ash content is less than 2%. Polylactic acid (PLA) pellets were purchased from Inzea F38 from Nurel biopolymers and it contains about 70% of biobased content with a density of 1.23 g/cm^3^ and melt flow index of 1.8 g/10 min. NaOH (reagent grade, ≥98% purity), Propylene carbonate (anhydrous, 99.7% purity), Pyridine (reagent grade, 99.8% purity), 2-chloro-4,4,5,5-tetramethyl-1,3,2-dioxaphospholane (TMDP), N-hydroxy-5-norbornene-2,3-dicarboxylic acid imide (NHND), chromium (III) acetyl acetonate (99,99%), 2,2′-Azino-bis(3-ethylbenzothiazoline-6-sulfonic acid) diammonium salt (ABTS) (≥98%), 2,2′-azobis (2-amidinopropane) dihydrochloride (AAPH) (99,8%), linoleic acid (analytical grade ≥99%), chloroform CDCl3 (99.9%) and N,N Dimethyl formamide DMF (anhydrous 99.99%) were purchased from sigma Aldrich.

### Lignin modification using oxypropylation

Kraft lignin (5 g) was first placed into a 250 ml round bottom flask. Then, 30 g of propylene carbonate and 0.133 g of NaOH were added to the lignin and stirred using a magnetic stirrer at 170°C for 3 h. After the reaction time, the oxypropylated lignin (OPKL) product was recovered through precipitation using acidified water (pH = 2), followed by filtration through cellulose membrane filters.

### Particle size and molecular weight analysis

Particle size distribution of lignin and oxypropylated lignin were measured using laser scattering particle size distribution analyzer (Horibo particle size analyzer LA-960V2). Particle size analyzer measured that mean particle size value of 63 µm for kraft lignin and 82 µm for oxypropylated lignin, respectively.

Malvern OMNISEC Resolve multi-detector instrument equipped with Two PLGel 10 µm Mixed B 300 mm × 7.5 mm columns and THF used as an eluent to investigate the molar weight distribution of polymers using size exclusion chromatography (SEC). Flow rate of 0.5 ml/min and 20 µL of kraft lignin (KL) and oxypropylated lignin (OPKL) samples with the concentration of 2 mg/ml were injected separately with the run time of 60 min. KL and OPKL were dissolved in THF solvent and filtered through 0.45 µm PTFE filters prior to the injection. SEC chromograms of KL and OPKL along with number average molecular weight (Mn), weight average molecular weight (Mw) and polydispersity index (Mw/Mn) are presented in Supporting Information Figure 2. The obtained SEC results showed that the weight average molecular weight (Mw) of KL and OPKL was found to be 5,702 g mol^−1^ and 4,876 g mol^−1^, respectively.

### PLA/lignin composite preparation

PLA/lignin composites were produced through melt blending followed by injection molding. PLA/kraft lignin (PLA/KL) and PLA/oxypropylated lignin (PLA/OPKL) were blended using a Thermo Scientific™ HAAKE ™ PolyLab OS Torque Rheometer with the operating condition of temperature at 170°C, rotation speed of 100 rpm and 8 min of mixing time. PLA with unmodified kraft lignin and oxypropylated kraft lignin blends were produced at weight percentages of 1, 5, 10, 20, and 40% lignin. The produced blends were ground using a Fritsch PULVERISETTE 25/19 cutting mill with a 4 mm sieve. After grinding, blends were injection molded into tensile samples using a Thermo Scientific™ MiniJet Pro with the holding temperature at 180°C and molding temperature at 80°C.

### NMR analysis

The hydroxyl group contents in KL and OPKL were analyzed by quantitative ^31^P NMR spectroscopy. 30 mg of oven-dried lignin (45°C, 24 h) was dissolved in 0.150 ml of DMF and 0.100 ml of pyridine. 2-Chloro-4,4,5,5-tetramethyl-1,3,2-dioxaphospholane (TMDP) (0.94 mmol, 0.150 ml) was used as a phosphitylating reagent, endo *N*-hydroxy-5-norbornene-2,3-dicarboxylic acid imide (NHND) (0.010 mmol, 0.200 ml) as an internal standard, and chromium (III) acetyl acetonate (0.050 ml) as a relaxation agent. Finally, 0.300 ml of CDCl_3_ was added. Fully soluble samples were transferred to NMR tubes, and the spectra were recorded within 1 h of the sample preparation. The ^31^P NMR experiment was performed with a 90⁰ pulse angle, inversed gated proton decoupling, and a delay time of 10 s. For the analysis, 256 scans with a delay time of 10 s and a total runtime of 30 min for each sample were used. Two replicated experiments were conducted, and the mean value with one standard deviation is reported (Moreno et al., 2021). Degree of substitution (DS) between was calculated using either phenolic hydroxyl content or similarly with carboxylic acids according to

$$DS=100\%∙\frac{Phenolic\;OH\;\left(KL\right)-Phenolic\;OH\;\left(OPKL\right)}{Phenolic\;OH\;\left(KL\right)}$$

where the contents of phenolic OH or carboxylic acid groups are in units mmol/g.

### Scanning electron microscopy (SEM)

The morphology of PLA lignin composite specimens was investigated by a field-emission scanning electron microscope (FE-SEM; Zeiss Sigma VP, Germany). To examine the surface morphological features of fractured surfaces obtained from the tensile test of the specimens were attached onto carbon tape. Then the samples were sputter-coated with gold under the operating condition of 60 s, 10 mA. Micrographs of surface were obtained at the accelerating voltage of 5 kV using a secondary electron detector.

### Thermogravimetric analysis (TGA)

Thermal stability of KL and OPKL samples were analyzed in Platinum pans using a Waters TA Instrument™ TGA 5500. The samples (5–10 mg) were tested in a temperature range from 40 to 600°C with a heating rate of 10°C min^−1^ under a nitrogen atmosphere with a flow rate of 25 ml min^−1^.

### Differential scanning calorimetric (DSC) analysis

DSC measurements of PLA and PLA/AKL bio composites were performed in Tzero Aluminum pans under a nitrogen atmosphere with a temperature range of −25–210°C at a heating rate of 10°C/min and held at 210°C for 2 min to remove the thermal history and then cooled to −25°C at the rate of 10°C/min and in the second heating scan again temperature increased up to 210°C with the heating rate of 10°C/min. Glass transition temperature (T_g_), cold crystallization temperature (T_cc_) and melting temperature (T_m_) were calculated from the recorded thermograms. Crystallinity of the samples were calculated from the thermograms from the second heating scan.

### Mechanical test

The mechanical performance of neat PLA and PLA with KL and OPKL composites was evaluated using a Zwick universal Test Machine. Injection-molded, dog-bone shaped composite specimens (width 5 and 2 mm thickness) were tested according to the ASTM D638 standard (Type IV) using a crosshead speed of 5 mm min^−1^ and an initial grip separation of 50 mm. Force and displacement were measured and recorded. A minimum of five composite specimens were measured to calculate Young’s modulus, tensile strength, and elongation at break values.

### Antioxidant test

Antioxidant properties of PLA/KL and PLA/OPKL composite specimens were recorded using a UV-Vis spectrometer in the range of 200–800 nm with air as the background. The antioxidant activity assay was adapted from Faroog et al. (Farooq et al., 2019). Freshly prepared ABTS radical cation solution was diluted (1:40) until it reached an absorbance of 0.7 at 734 nm at 25°C. Then 2 mg of specimens were immersed in vials containing 2 ml of ABTS radical cation solution and agitated in a thermostatic orbital shaker at 25°C. The ABTS^•+^ assay and the radical neutralization could be attained through a direct reduction *via* electron transfer or radical transfer *via* hydrogen atom (García et al., 2012). Then the radical scavenging activity of the specimens was monitored through the time evolution of the absorbance at 734 nm.

### Lipid-peroxidation study

The lipid peroxidation study was performed to monitor the capability of the PLA/KL and PLA/OPKL activity for storing lipid-containing food substances. The antioxidant activity of PLA/KL and PLA/OPKL were measured with a response at 234 nm in UV (Liégeois et al., 2000). When the lipid undergoes oxidation, conjugated diene hydroperoxide is produced, which is detectable at the wavelength of 234 nm using a UV-Visible spectrophotometer. Substrate solution (30 μL; 16 mM linoleic acid) and 10 mg of PLA/KL and PLA/OPKL specimens were added to 2.81 ml of 0.05 M phosphate buffer (pH 7.4), which was previously thermostabilized at 37°C. Then, 150 µL of 40 mm concentrated AAPH solution was added. The progress of oxidation was measured by recording the increase in absorbance at 234 nm *versus* a blank cuvette containing the same reaction mixture except for the substrate solution (Peyrat-Maillard et al., 2003).

### Overall migration test

Overall migration activity of PLA/KL and PLA/OPKL was carried out to account for the application of produced composites for fresh food packaging. The analysis was performed by immersing 300 mg of the samples in 5 ml of ethanol:water (1:9 v/v) solvent mixture. After 10 days of gentle agitation at 29°C, the absorbance from the liquid phase was measured using a UV-visible spectrometer (Yang et al., 2016).

## Results and discussion

### NMR analysis of crude and oxypropylated kraft lignin

The goal of this study was to evaluate whether hydroxypropylation improves compatibility and multifaceted properties of PLA-based composites that are considered for food packaging applications.

**FIGURE 1:**
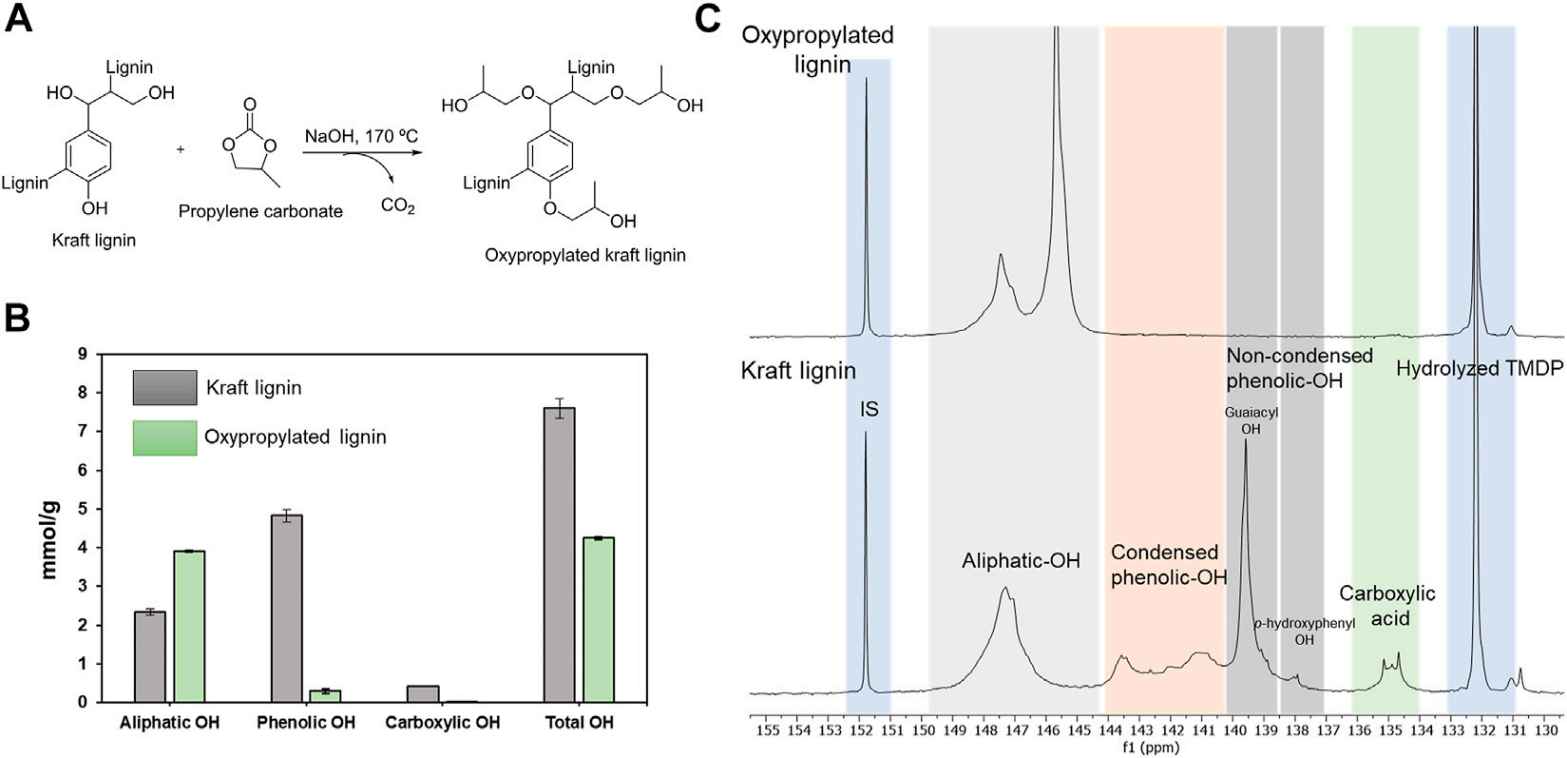
**(A)** Schematic reaction of the oxypropylation of kraft lignin. **(B)** Distribution of aliphatic hydroxyl, carboxylic acid and phenolic hydroxyl groups in kraft lignin and Oxypropylated lignin. Error bars show the standard deviation from the average values obtained from two replicates of the quantitative ^31^P NMR experiments. **(C)** ^31^P NMR spectra of Oxypropylated kraft lignin (top) and kraft lignin (bottom).

Oxypropylation reaction of lignin was performed to alter the chemical structure of the lignin and attempted to enhance the compatibility with PLA. This reaction introduces a new aliphatic side chain, which is anticipated to improve plasticity and mobility of the modified lignin. To analyze the efficiency of the derivatization reaction the original kraft lignin and the oxypropylated lignins were first subjected to quantitative ^31^P NMR analysis. The oxypropylation converted the hydroxyl groups, including both phenolic and aliphatic hydroxyl groups into etherified lignin with aliphatic hydroxyl groups at the end. The oxypropylation reaction scheme is shown in Figure 1A. A comparison of quantitative ^31^P NMR results showed that phenolic hydroxyl groups and carboxylic acid functionalities were effectively reacted by oxypropylation (Figure 1B). Compared to the contents of kraft lignin, these two functionalities allow estimating a degree of substitution between 93 and 94%. The increase in aliphatic hydroxyl content by 67% can be explained by the competition of reaction between aliphatic and phenolic hydroxyl groups, with the latter contributing to the increase of aliphatic OH content.

The results of the ^31^P NMR analysis indicated that two broad signals appeared between 144.5 and 149.5 ppm, corresponding to the aliphatic hydroxyl groups (Figure 1C) (Sadeghifar et al., 2012). Specifically, the signal at 147.5 ppm represents the primary and secondary aliphatic hydroxyl groups, which are initially present in the kraft lignin (before modification), and the new signal appeared at around 145.5 ppm, corresponding to the newly formed aliphatic hydroxyl groups that are introduced through the oxypropylation process. Based on the obtained results, it was apparent that the oxypropylation reaction was successful and the original and modified lignins were used to prepare injection molded PLA composites.

### Morphological characterization

**FIGURE 2:**
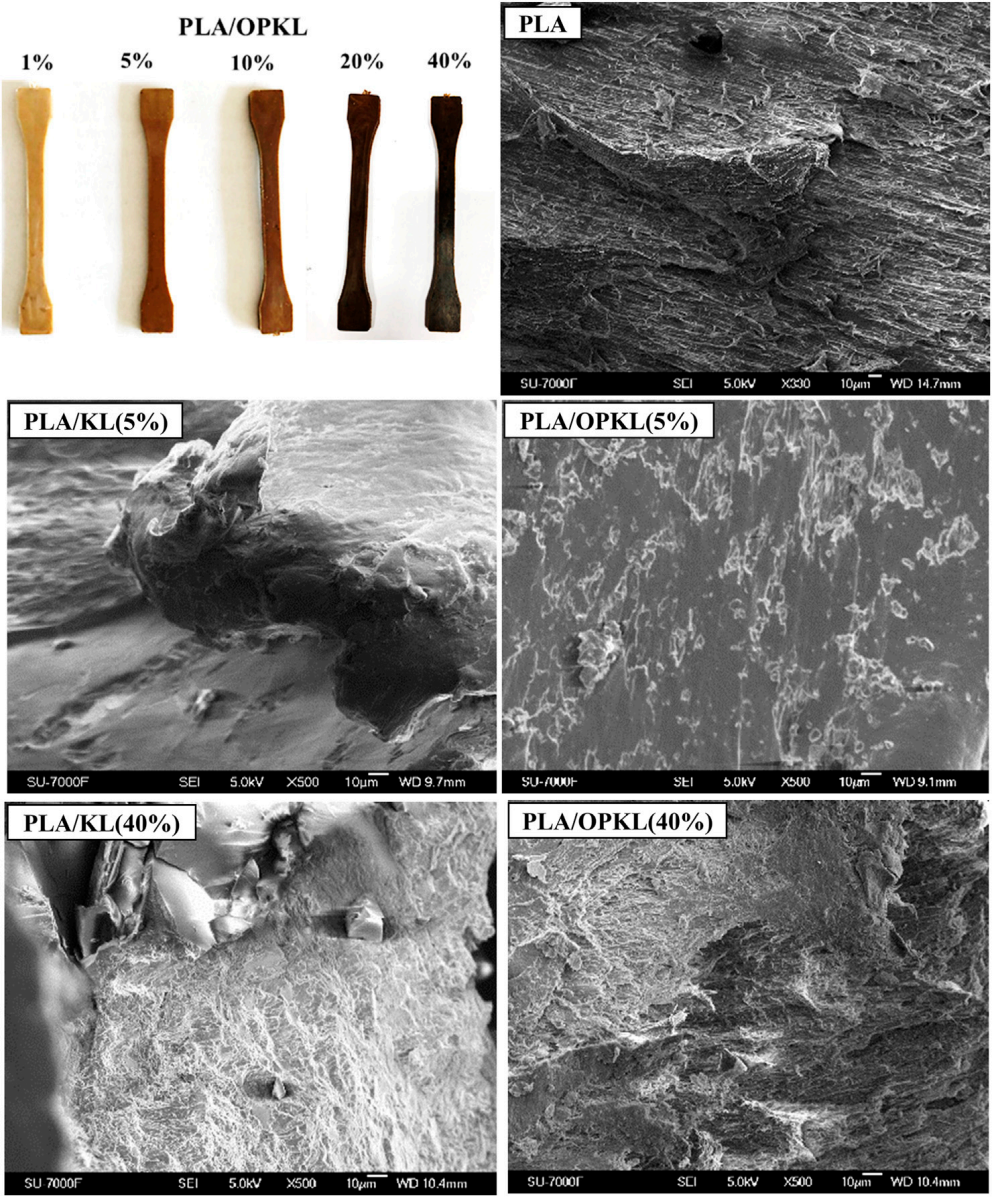
Injection molded PLA/OPKL composites specimens (top-left) and the SEM micrographs of neat PLA, PLA/KL, and PLA/OPKL tensile-fractured surfaces with different lignin and oxypropylated lignin contents. The scale bars in SEM images: 10 μm.

Morphological investigation of polymeric composites provides important information about compatibility of the filler and the matrix components. The injection molded specimens are shown in Figure 2 with different lignin wt% and it can be anticipated that increasing wt% of lignin content in PLA matrix logically increased brown color in the composites. The results of the SEM analysis provided the distribution of the lignin particles in the polymer matrix. Morphology of PLA/KL and PLA/OPKL composites were investigated to account for the compatibility and arrangement of the lignin in PLA matrix. The fractured surfaces from the broken tensile specimens revealed the brittleness of neat PLA as the samples exhibited a smooth morphology (Figure 2). With 1% of KL addition in PLA (Supplementary Figure S1), the fracture morphology indicated a rough surface and an increase in elongation. With increasing KL content to 5%, no traces of substantial elastic behavior can be seen, and which is also consistent with the result obtained for the elongation at break values (Figure 5B). Upon increasing the KL content up to 40%, the surface appeared rougher and was attributed to the poor compatibility of lignin and its aggregation behavior within the composites. Conversely, the PLA/OPKL composites displayed a considerably different morphology compared to that of PLA/KL. The inclusion of OPKL in PLA enhanced the smoothness of the fractured surface, which clearly indicated the improved compatibility of oxypropylated lignin in PLA. The improved compatibility most likely enhanced the interfacial strength between OPKL and PLA. For instance, PLA/OPKL (1 and 5%) showed a smoother surface compared to PLA/KL due to improved adhesion, which clearly explained the plasticity character of OPKL. As seen in Supplementary Figure S1 the case of 10% of OPKL in PLA illustrated the presence of an extended elongation in the surface with some cavities that showed the possibilities of holding the OPKL and PLA together and enhancing the plastic deformation during tensile tests. However, higher OPKL content (20 and 40%) greatly induced toughness of the materials that cannot possess the elongation behavior and led to instant failure. Therefore, the investigation of morphological analysis of unmodified and modified lignin with PLA composites concluded that the introduced oxypropyl chain’s reaction increased the homogenization of lignin in the system, which subsequently contributed to the plasticity of the entire system. In comparing the results obtained from elongation at break and SEM micrographs, the analysis indicated that 5% OPKL could potentially enhance the compatibility with PLA. However, the higher percentages of the lignin in PLA result in poor ductility.

### Thermogravimetric analysis

**FIGURE 3:**
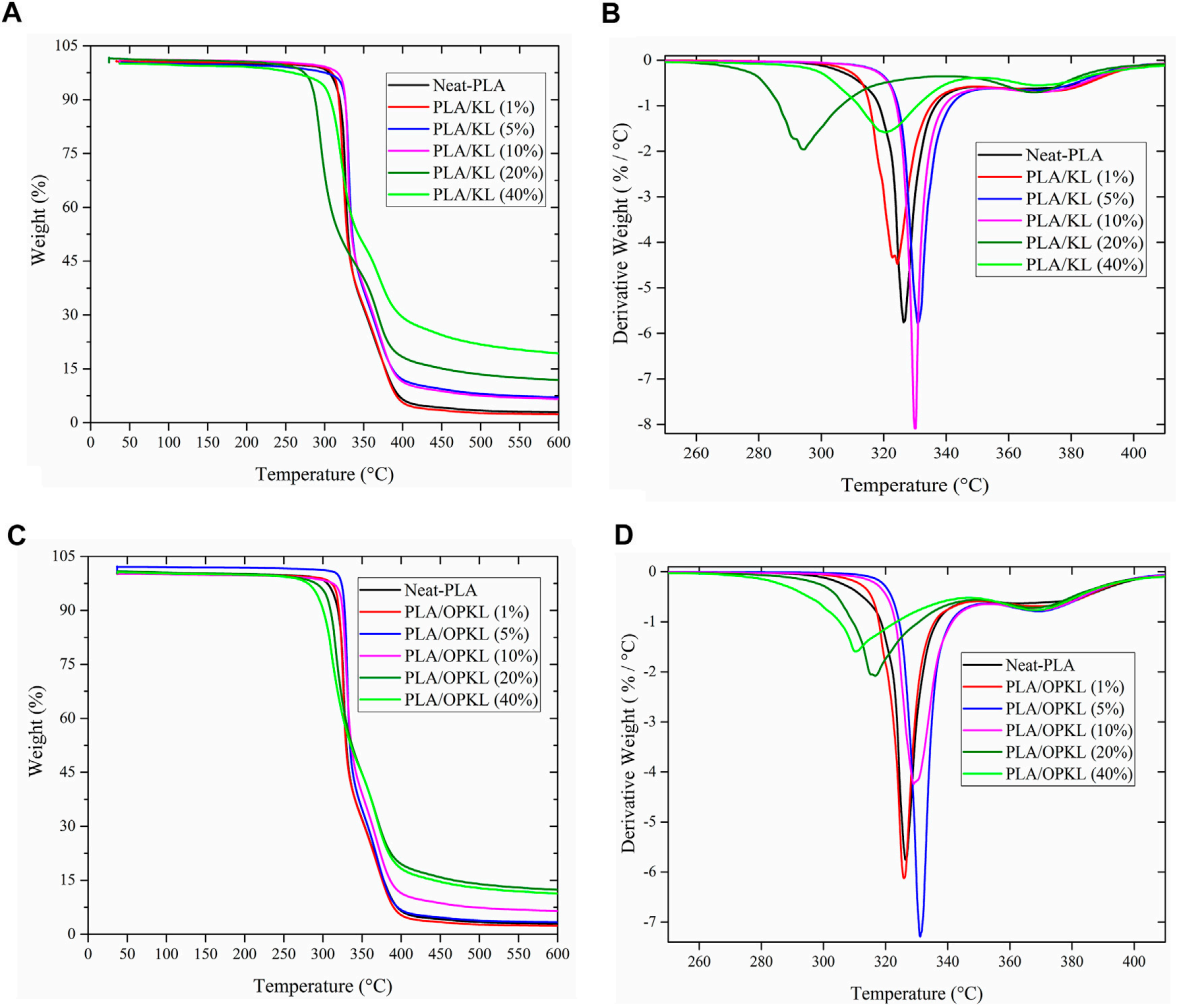
TG and the corresponding DTG curves of **(A,B)** PLA/KL and **(C,D)** PLA/OPKL composites materials.

Thermal stability is an important characteristic used to evaluate composite materials for specific end-use applications. Understanding the thermal stability of PLA/lignin composites is important because melt processing might cause degradation and emission of volatile chemicals of concern in food packaging. Thermogravimetric curves and first derivative thermogravimetric curves for neat PLA, PLA/KL and PLA/OPKL composites are shown in Figure 3, while Table 1 provides the results for the temperature at which the composites start to degrade (T_onset_), and when thermal degradation is completed (T_offset_). Thermal degradation of neat PLA starts at 323°C (T_onset_) and completes at 340° (T_offset_). With the addition of KL at 1%, the T_onset_ started earlier than with neat PLA. However, in samples with 5 and 10% of lignin content, initial degradation started later than with neat PLA while still ending at a similar temperature. When increasing the KL weight percentage to 20 and 40%, the material began to degrade at a lower temperature of 282°C. As reported in another study, the low molecular weight compounds of lignin could cause such a decrease in the degradation temperatures (Cui et al., 2013). For PLA/OPKL blends with 1 and 5%, T_onset_ occurred at 326°C, which was 3°C higher than that of neat PLA. When the OPKL content increased to 10% and higher, the degradation started earlier. However, the degradation ended (T_offset_) at higher temperatures compared to neat PLA and PLA/KL. In the present work we found that the percentage of the residue after the thermal degradation process increased with increasing lignin content. This result likely occurred given the lignin’s aromatic structure and its strong ability to produce char (Zhang et al., 2012). Neat PLA left a char residue of 2.8% at 600°C, whereas the lignin and oxypropylated lignin/PLA blends at 10% showed a char residue of around 6%, and for those at 40% of lignin content, the char residue was around 17%. Increasing oxypropylated lignin percentage in PLA composites could be advantageous as lignin char residue has been reported to reduce the combustion heat and heat release, and because of such characteristics, lignin is used as a flame-retardant additive in composites (Watkins et al., 2015; Park et al., 2019). Overall, the results indicated that PLA/OPKL composites showed slightly improved thermal stability as compared to PLA/KL composites and neat PLA.

**TABLE 1:** Thermal properties of neat PLA, PLA/KL and PLA/OPKL composites.

	Lignin content (%)	T_onset_ (°C)	T_offset_ (°C)	T_max_ (°C)	Residual wt% at 600°C
Neat PLA		323.6	340.8	330.2	2.84
PLA/KL	1	316.9	339.7	324.3	2.21
	5	326.0	342.6	331.1	6.43
	10	326.1	339.0	329.5	6.06
	20	282.6	331.1	294.0	10.50
	40	306.1	354.2	320.7	17.64
PLA/OPKL	1	325.1	339.0	329.2	1.47
	5	326.9	341.0	332.0	3.09
	10	323.6	346.0	329.0	5.97
	20	307.0	349.3	317.2	11.30
	40	296.1	358.3	311.5	10.24

T_onset_—Starting temperature of the degradation process.

T_offset_—degradation temperature of completed process.

T_max_—The maximum degradation temperature of the composites.

Residual wt% at 600°C—The percentage amount of char residue obtained at the end of the degradation process.

### Differential scanning calorimetry (DSC) analysis

One important issue related to PLA in composite applications is its poor crystallization behavior, and thus it is important to understand changes in thermal properties and material responses when incorporating lignin. DSC analysis was performed to investigate the influence of different weight percentage of KL and OPKL in the PLA matrix. Specifically, the T_g_, T_cc_ and Tm values were used to analyze the potential effectiveness of the composites for the packaging sector. DSC curves of kraft lignin and oxypropylated kraft lignin were given in supporting information Figure 4. Tg of kraft lignin was around 95°C and oxypropylated kraft lignin was 84°C.

**FIGURE 4:**
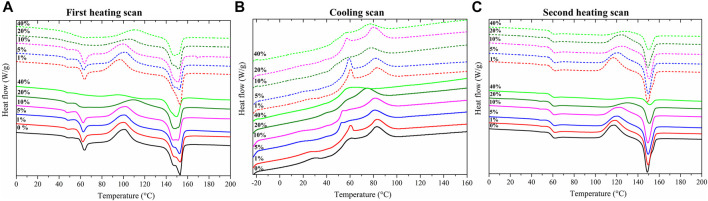
DSC curves of **(A)** first heating scan, **(B)** cooling scan and **(C)** second heating scan. **(A–C)**: Continuous lines—PLA + *X* % of KL and Dashed lines—PLA + *X* % of OPKL. (*X* is 1, 5, 10, 20, and 50%).

Compared to neat PLA, all PLA/KL and PLA/OPKL composites showed similar or slightly lower glass transition temperature (T_g_) values, which could be due to the plasticizing effect of low molecular weight fractions of lignin (Figure 4A). The substitution of the phenolic hydroxyl groups by aliphatic hydroxyl groups using oxypropylation is expected to decrease the T_g_ of the oxypropylated lignin (Cui et al., 2013). After the modification, the hydrogen bonding interaction in the original kraft lignin could have been decreased, leading to an increased free volume of the polymer system, which is reflected in the reduction in T_g_ value. In the thermogram shown in Figure 4A, after T_g_ curve, the first peak corresponds to the cold crystallization peak (range between 95 and 115°C), followed by the second endothermic peak, which indicates the melting-recrystallization-melting peak of PLA. The two melting peaks obtained from the lignin-PLA endothermic process show that the first melting peak is associated with the less perfect PLA crystal, and the second peak denotes a more perfect PLA crystal and these phenomena are consistent with the reported work of Mu et al. (Mu et al., 2014). Upon addition up to 10 wt% in PLA of KL and OPKL there was an improved cold crystallization temperature, as T_cc_ values were shifted from 102.6 to 100°C (Table 2). A similar trend was observed in research by Kim et al. who found that the introduction of lignin or acetylated lignin introduced heterogeneous nucleation and decreased the T_cc_ value of the composites (Kim et al., 2017). Above 10% of KL content, PLA’s crystallization rate decreased, which led to the shift in cold crystallization values to a higher temperature as the T_cc_ value shifted above 102.6°C. The exothermic peak of cold crystallization decreased considerably at the higher wt% of KL and OPKL (40%). This decrease was most likely due to the restricted molecular motion of PLA chains by lignin and modified lignin. Considering the melting properties, up to 20% addition of OPKL favored the crystallization and increased the intensity of the T_m2_ peak (Figure 4A), whereas, in the case of PLA/KL, these phenomena were observed only until 10% of KL. However, no distinct behavior was observed for melting peaks in the thermograms for 40% of KL. These results indicated that lignin addition improved the cold crystallization temperature of PLA, which was our expectation for improving the PLA/Lignin composite properties, in which OPKL improved T_cc_ values consistently until a weight fraction of 10% and showed the improvement in the T_cc_ values compared to unmodified lignin addition.

**TABLE 2:** DSC parameter values of neat PLA, PLA/KL and PLA/OPKL composites: T_g_—glass transition temperature, T_cc_—cold crystallization temperature and T_m1_ and T_m2_—melting temperatures from first heating scan.

	Lignin (%)	DSC values (°C)							
		First scan				Second scan			
		T_g_	T_cc_	T_m1_	T_m2_	T_cc_	T_m_	ΔH (J/g)	χ^α^
Neat PLA		60.3	102.6	147	153	117.3	148.5	16.30	17.52%
PLA/KL	1	58.5	100.5	148	153	118.8	149.5	18.51	20.10%
	5	58.1	101.2	146	153	120.7	149.6	14.14	16.00%
	10	58.4	101.8	147	151	122.8	149.4	12.71	15.18%
	20	57.0	109.6	147	151	130.8	150.8	7.68	10.32%
	40	59.7	96.6	-	150	134.5	151.5	2.25	4.03%
PLA/OPKL	1	60.5	100.3	147	153	117.5	148.8	17.57	19.08%
	5	59.6	101.9	147	152	121.4	149.8	15.61	17.67%
	10	59.9	100.4	147	151	125.6	150.6	12.95	15.48%
	20	52.9	105.9	147	152	124.9	149.5	9.39	12.63%
	40	56.8	111.0	148	152	127.8	150.3	3.21	5.75%

Crystallinity χ ^α^ = (∆H/∆H*)/Φ_PLA_; ΔH, is the melting enthalpy calculated by integrating the melting peak in the DSC, second heating scan shown in Figure 4A; ∆H* = 93 J g^−1^ is the melting enthalpy of a 100% crystalline PLA; and Φ_PLA_, is the weight fraction of PLA, in the samples (Zhang et al., 2018).

By looking at the cooling scan, it was clearly seen that at higher KL and OPKL content, the crystallization peak completely disappeared (Figure 4B). This absence of crystallization indicates that higher weight fractions of amorphous lignin were thoroughly blended with PLA at a molecular level. Another interesting aspect is the effect of lignin on the cold crystallization temperature. As recorded from the second heating scan, in both cases PLA/KL and PLA/OPKL composites, there was an increase in the T_cc,_ indicating that the segmental motion of PLA chains was restricted by lignin (Figure 4C). At higher lignin contents of 20 and 40% the crystallization capacity has been affected, leading to the shift in cold crystallization temperature from 117 to 134°C and 117–127°C for the PLA/KL and PLA/OPKL, respectively (Table 2). These shifts in cold crystallization temperature confirmed that at higher lignin content it is preferable to incorporate OPKL rather than unmodified KL.

### Mechanical properties

PLA has reasonably high tensile strength and modulus, but its high cost (about 5140 US$/per ton (Ratshoshi et al., 2021)) and limited thermal stability are the major factors limiting its use in food packaging applications. One way to potentially decrease the cost is to add lignin as a filler in PLA.

**FIGURE 5:**
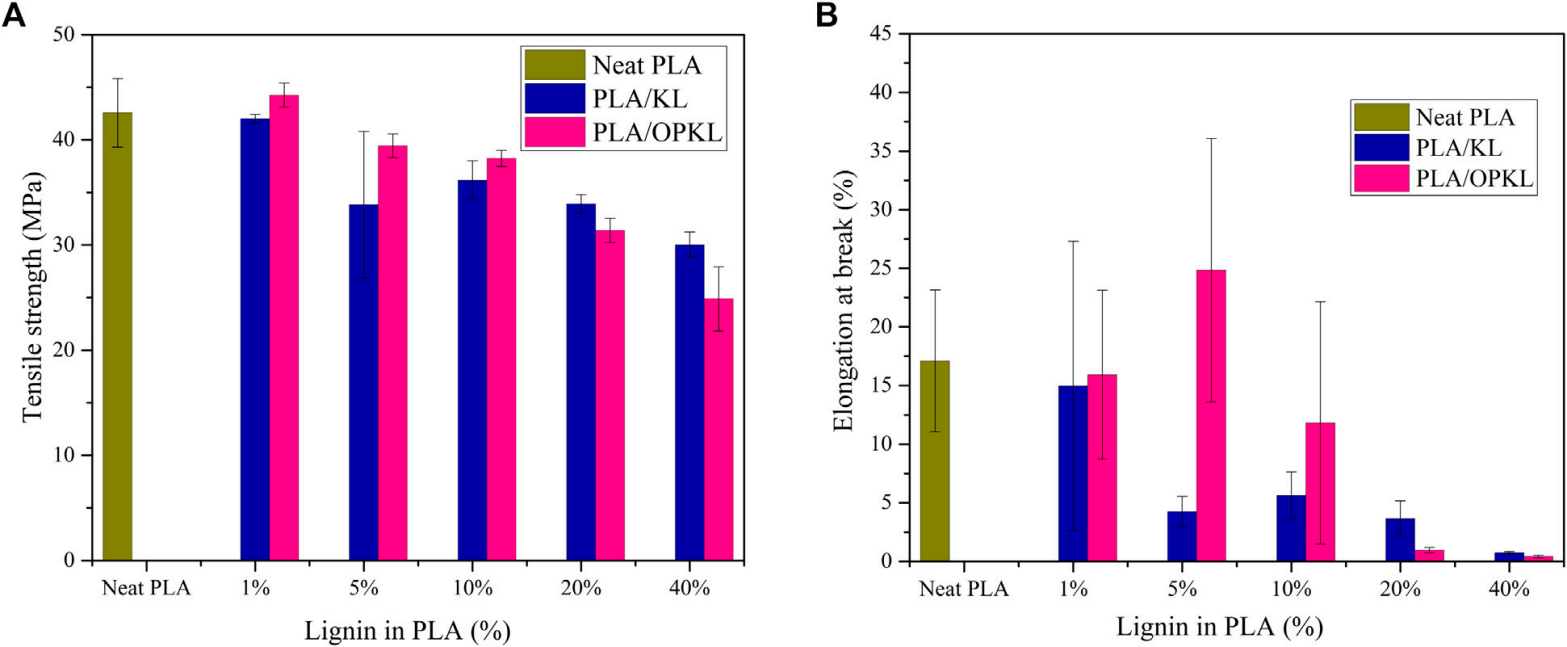
Mechanical properties of KL/PLA and PLA/OPKL, **(A)** Tensile strength, and **(B)** Elongation at break.

The Young’s moduli of each composite blends are shown in Supplementary Figure S2 and tensile strength and elongation at break are demonstrated in Figure 5. PLA exhibited a tensile strength of 42.6 MPa and a modulus of 2,120 MPa. From this starting point, the incorporation of increasing amounts of KL or OPKL generally did not have a significant impact on modulus. In contrast, the tensile strength of composites with OPKL contents up to 5% was comparable to that of PLA. However, the tensile strength decreased at increased lignin contents. A key observation was that the composite with OPKL performed systematically better than those with unmodified KL, with the exception of 20 and 40% lignin contents. Similar observations were previously made with PLA-pristine and PLA-acetylate lignin at 1, 5, and 10% (Kim et al., 2017). This trend can also be evidenced by the values obtained through the elongation at break measurements. Compared to neat PLA, PLA/OPKL at 5% addition exhibited a higher percentage of elongation at break. After 5% of lignin addition, the tensile strength and elongation at break point were reduced. The reason is that the interaction between the stress concentration zones around lignin particles were getting closer at higher lignin content which leads to reduced mechanical properties (Kargarzadeh et al., 2020).

Comparing PLA/KL with PLA/OPKL, elongation at break at 1, 5, and 10% were higher for PLA/OPKL. Most likely, the low molecular weight fractions of lignin act as a plasticizer and increase the ductility of the composite. In the modified lignin, the introduced aliphatic hydroxyl groups further improve the ductility to 10% PLA/OPKL. In the case of 20 and 40% of PLA/KL showed a very low elongation at break, which indicated increased brittleness of the material. Similar trends in tensile strength and elongation at break were observed with 20 and 40% PLA/OPKL.

Young’s modulus increased with increasing OPKL content of 5, 10, and 40% with PLA. In the case of PLA/KL, Young’s modulus increased already at 1% KL addition compared to neat PLA, whereas at 10 and 20% KL contents the obtained Young’s moduli were in the range of neat PLA. In all cases, these overall tensile strength reductions can be due to lignin and modified lignin tensile strength alone being lower than neat PLA. For this reason, oxypropylation does not necessarily improve the overall tensile strength even if the lignin becomes better miscible in the blend. Agglomeration of lignin is also a probable cause of lower tensile properties at higher percentages.

### Antioxidant test

**FIGURE 6:**
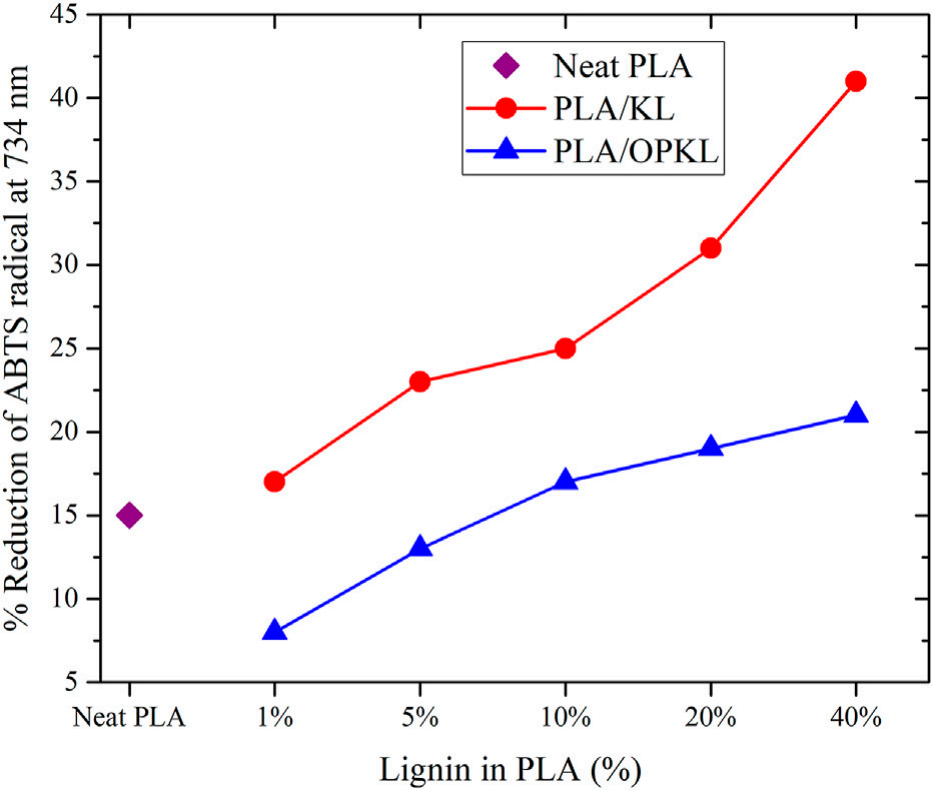
Reduction of ABTS^•+^ radical of Neat PLA, PLA/KL and PLA/OPKL at 734 nm.

Lignin consists of free phenolic groups that provide favorable possibilities for use as radical scavengers in food packaging materials. The antioxidant activity of PLA/KL and PLA/OPKL composites was monitored using ABTS^•+^ radical scavenging method (García et al., 2012). The reduction in absorbance of ABTS^•+^ at 734 nm was studied in the presence of PLA/KL and PLA/OPKL composites. The measured values of all examined composites are shown in Figure 6, along with comparisons to neat PLA’s reduction ability. PLA/KL showed higher antioxidant activity over those of PLA/OPKL composites. The relative values of antioxidant activity of PLA/KL increased stepwise from 17 to 42% as the lignin content of the composite increased. The NMR results (Figure 1) indicated that during the oxypropylation reaction, the active phenolic hydroxyl groups were converted to ether linkages and increased the content of aliphatic hydroxyl groups. This conversion decreased their antioxidant activity. Prior studies have evidenced that lignin’s antioxidant activity depends on various parameters, but mainly the content of phenolic hydroxyl groups and the molecular weight (Dizhbite et al., 2004; Pan et al., 2006; Ugartondo et al., 2008). Several authors (Cai et al., 2004, 2006; Aadil et al., 2014) reported that the free radical scavenging and antioxidant activity are mainly attributed to the number and position (ortho dihydroxy phenolic moieties) of the hydrogen donating hydroxyl groups of the phenolic molecules.

The radical scavenging activity is an important process that majorly inhibits free radicals in food packaging materials. The antioxidant test results revealed that OPKL is capable of reducing the radicals in the food packaging. 5% of OPKL exhibited almost the same percentage of scavenging activity as PLA. However, additional studies such as lipid oxidation and migration analysis are necessary to evaluate suitability of PLA-lignin composites for food packaging applications.

### Lipid peroxidation assay

**FIGURE 7:**
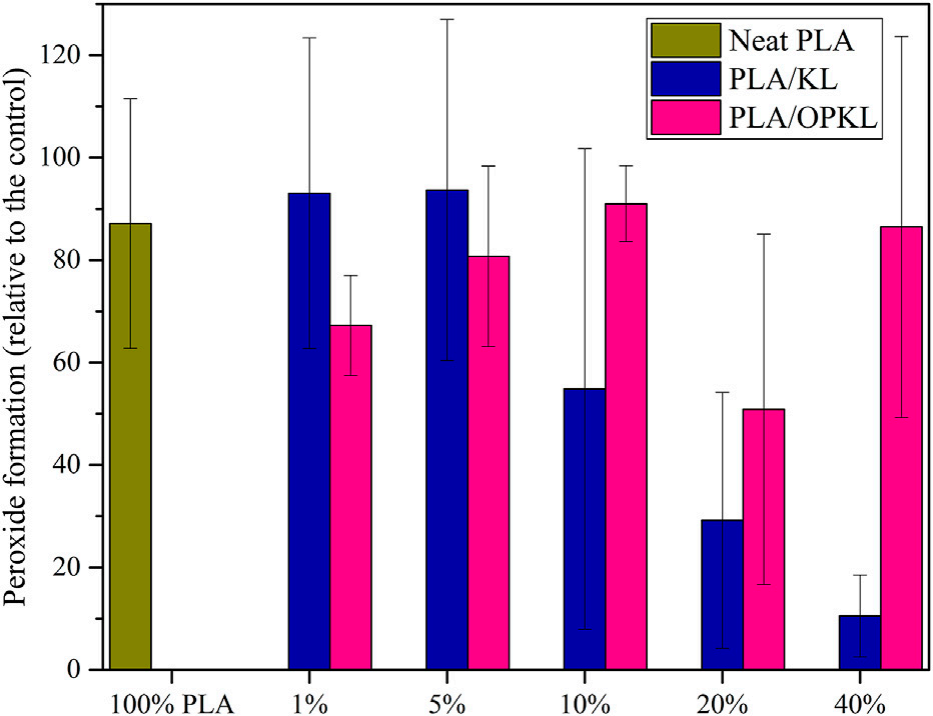
Antioxidant results of neat PLA, PLA/KL and PLA/OPKL with respect to the control at 234 nm in UV.

Lipid oxidation is a vital issue that greatly deteriorates food containing significant content of lipids such as nuts, oils and meats (Gómez-Estaca et al., 2014). The lipid oxidation can be prevented by including antioxidant compounds in the food itself or using antioxidant materials in the packaging used for lipid-containing foods. As seen in the previous section, KL and OPKL exhibited antioxidant characteristics in terms of radical scavenging activity. Therefore, these PLA/KL and PLA/OPKL were subjected further to evaluate their lipid antioxidant characteristics. The lipid-based antioxidant activity was carried out with 2,2′-azobis (2-amidinopropane) dihydrochloride (AAPH) as an oxidation initiator to elucidate lignin’s ability to inhibit fatty acid (linoleic acid) peroxidation. When linoleic acid undergoes oxidation in the presence of AAPH, it produces a conjugated diene hydroperoxide that can be quantified from its UV absorbance at 234 nm (Peyrat-Maillard et al., 2003). The addition of lignin is expected to inhibit formation of the conjugated diene by scavenging the free radicals in the system. Figure 7 shows antioxidant activity of lignin results after 3 h of reaction time. The control sample without lignin typically showed higher absorption at 234 nm, whereas especially unmodified KL inhibited the formation of linoleic acid hydroperoxide. However, in agreement with the antioxidant activities assayed with ABTS radical cation, OPKL had only a minor inhibitory effect on lipid peroxidation. The ability of lignin to prevent lipid peroxidation in cellulose based film materials has been reported before (Aguié-Béghin et al., 2015).

As seen in Figure 7, PLA/KL at 1 and 5% lignin content exhibited a minor extent of inhibition, which indicated that there was a threshold lignin content below which no substantial improvement in antioxidant properties occurs. The increasing KL content in PLA significantly increased the ability of the composites to inhibit lipid peroxidation, which was likely due to the phenolic hydroxyl groups.

Several research works and reviews have been conducted on the influence of fillers in the PLA matrix. There are two types of fillers majorly employed which include both organic and inorganic fillers such as polysaccharide nanocrystals (cellulose, chitin and starch) (dos Santos et al., 2017; Scaffaro et al., 2017), natural fibers (Mukherjee and Kao, 2011; Fiore et al., 2014), carbon fibers and carbon nanotubes (Papageorgiou et al., 2010), talc and hydroxyapatite (Liu et al., 2014) and organomontmorillonite (Bouakaz et al., 2015). The main objective of the reported fillers is to enhance thermal and mechanical properties and improve the crystallization of PLA. It is important to stress that the proposed fillers in the literature either enhance the thermal properties and decrease the mechanical properties and *vice versa*. For instance, the addition of natural fibers increased the flexural properties, but in contrast, tensile strength and the flexural strength were decreased compare to neat PLA (Fiore et al., 2014). Similarly, inorganic fillers (talc and hydroxyapatite) improved toughness of the PLA with decreased tensile strength and crystallinity (Liu et al., 2014). Therefore, comparing a broad range of existing fillers, the proposed oxypropylated lignin (OPKL) in this study has increased the crystallinity and mechanical strength of PLA in a lower addition (1 and 5%) and no major changes in the Tg values. Furthermore, these PLA/OPKL composites showed the optimum characteristics in antioxidant, lipid oxidation and migration studies.

### Migration test

**FIGURE 8:**
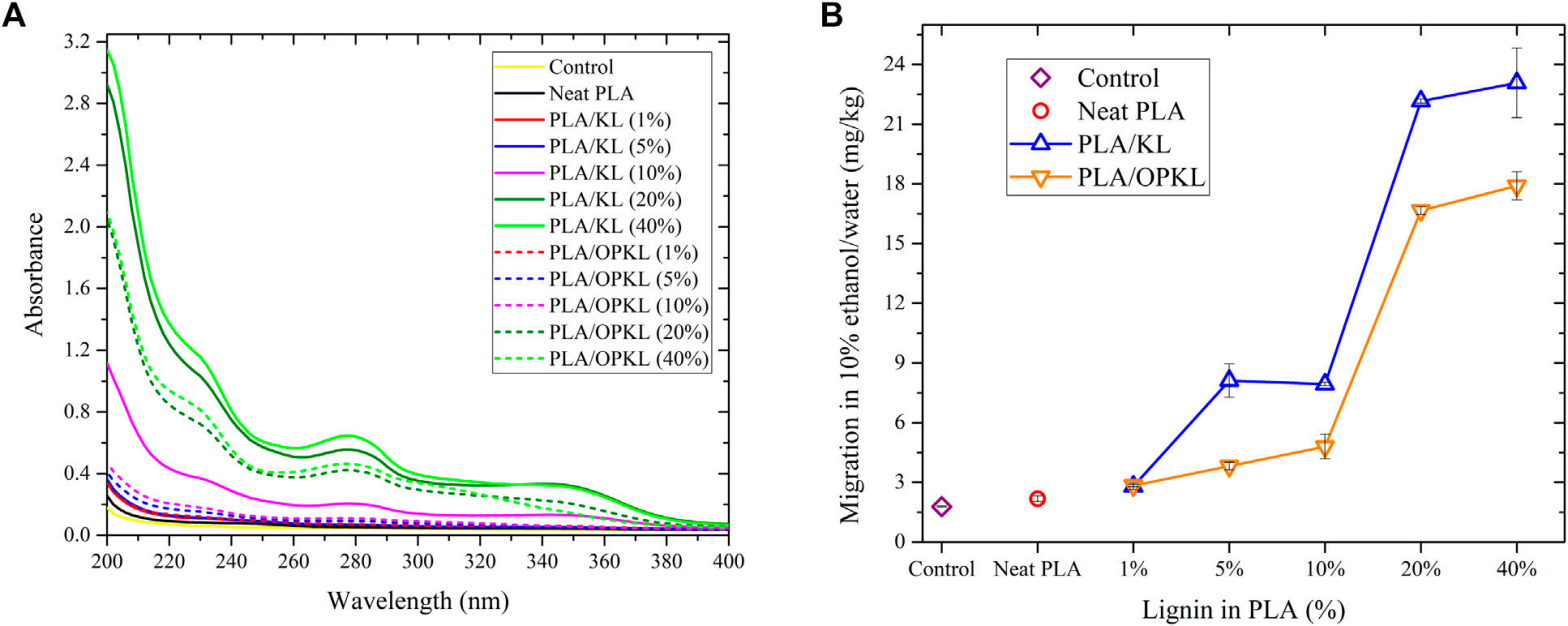
Migration study results, **(A)** Absorption of samples (control, neat PLA, PLA/KL and PLA/OPKL), and **(B)** overall migration of lignin content in 10% ethanol/water.

Migration tests were performed to evaluate suitability of the PLA-lignin composites for food packaging and to understand the correlation between anti-oxidant and lipid-peroxide inhibition. The performance of the PLA/KL and PLA/OPKL composite were investigated with a solution of 10% ethanol in water (Yang et al., 2016). For the PLA/KL and PLA/OPKL composites containing 1% lignin, there was a negligible amount of lignin leached, as shown by absorbance in the UV range (Figure 8A). Increasing lignin content revealed differences between KL and OPKL. Specifically, due to the free phenolic hydroxyl groups, unmodified KL, and better compatibility of OPKL in PLA, unmodified KL was expectedly more soluble in the aqueous ethanol extraction solvent. Therefore, systematically higher UV absorbance levels were recorded from the leachates originating from PLA/KL composites compared to the ones from PLA/OPKL. When calculated based on the absorbance at 280 nm and using an arbitrary extinction coefficient of 25 L g^−1^ cm^−1^ (Farooq et al., 2019), quantitative evaluation of the migratory substances can be performed. As shown in Figure 8B, the lignin-derived migratory substances remained at levels below 8.5 mg kg-1 when the lignin content was 10% or less. According to the current European legislation for food packaging material, the overall migration limit for food contact material is 60 mg kg^−1^ (Yang et al., 2016). Finally, the obtained values from this study have shown that the migration values of KL and OPKL are below the regulatory threshold of European legislation; therefore, the synthesized composites can be applied as packaging materials for food.

## Conclusions

In this work, we have synthesized and systematically characterized poly (lactic acid)/lignin composites using unmodified lignin and oxypropylated lignin in view of food packaging applications. The ^31^P NMR results confirmed effective oxypropylation of lignin with 94% degree of substitution of the phenolic hydroxyl groups to introduce new aliphatic hydroxyl groups. The 5% PLA/OPKL composite exhibited several benefits of the blending such as an improved cold crystallization temperature along with good mechanical properties with the higher elongation at break values and ductility compared to the pristine PLA. Furthermore, the composite formulations with up to 10% of oxypropylated lignin showed comparable performance with pristine PLA with respect to tensile strength, which further explains that oxypropylated lignin is a plausible candidate for sustainable PLA/lignin composites with added functionality such as protection against ultraviolet light in food packaging materials. However, there was a tradeoff between antioxidant activity and oxypropylation due to the loss of free phenolic hydroxyl groups. Nevertheless, lipid-oxidation and migration results indicated 5% PLA/OPKL as the most promising candidate for the preparation of food packaging composites. Overall, the result from this study suggests partially oxypropylated lignin as a plausible filler to tailor the properties and functionality of PLA-based composites for food packaging applications.

## Data Availability

The original contributions presented in the study are included in the article/Supplementary Material, further inquiries can be directed to the corresponding authors.
