# Supplementary material for: Multifunctional lignin-poly (lactic acid) biocomposites for packaging applications
Source: Front Bioeng Biotechnol. 2022 Oct 3;10:1025076. doi: 10.3389/fbioe.2022.1025076 (PMC9574040; doi:10.3389/fbioe.2022.1025076)
Supplement: Supplementary file 1 [file DataSheet1.PDF]

Supplementary Material

Multifunctional Lignin-Poly (lactic acid) Biocomposites for Packaging Applications

Esakkiammal Sudha Esakkimuthu[1*], David DeVallance[1,2*], Ievgen Pylypchuk[3], Adrian Moreno[3], Mika H. Sipponen[3*]

[1]InnoRenew CoE, Livade 6a, 6310 Izola, Slovenia

[2] Department of Materials and Environmental Chemistry, Stockholm University, Svante Arrhenius väg 16C, SE-106 91 Stockholm, Sweden

[3] University of Primorska, Faculty of Mathematics, Natural Sciences and Information Technologies, Glagoljaška 8, 6000 Koper, Slovenia

*** Correspondence:**
sudha.esakkimuthu@innorenew.eu
mika.sipponen@mmk.su.se
devallance@innorenew.eu

Contents

Supplementary Figures S1-S4

Supplementary Table S1

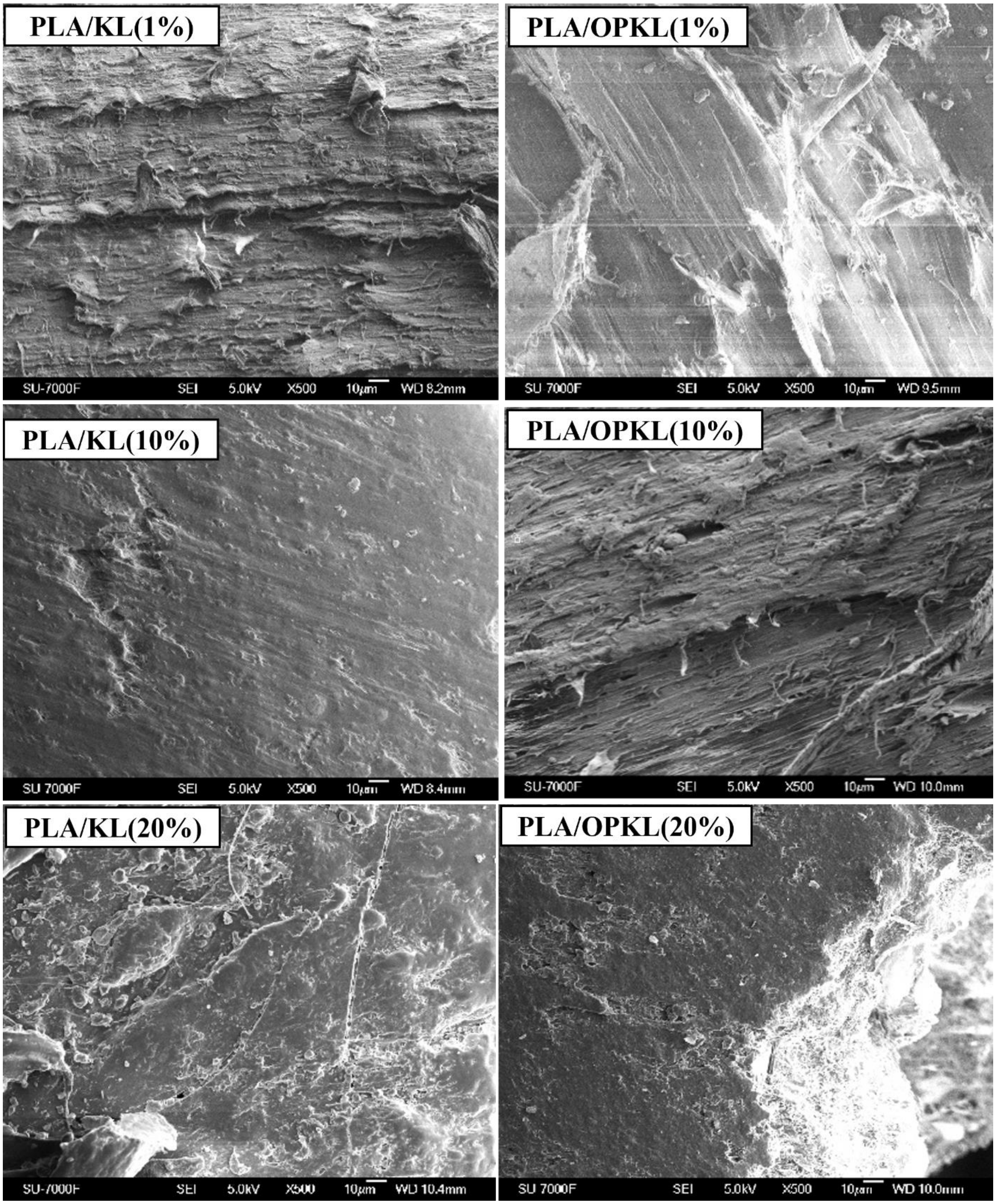

Figure S1. The SEM micrographs of neat PLA, PLA/KL and PLA/OPKL tensile-fractured surfaces

Size exclusion chromatography (SEC)

SEC analysis of kraft lignin and oxypropylated kraft lignin was performed using Malvern Omnisec resolve multi-detector system. The SEC chromatogram is presented in Figure S2 which is plotted between retention volume (vs) relative abundance from RI detector response.

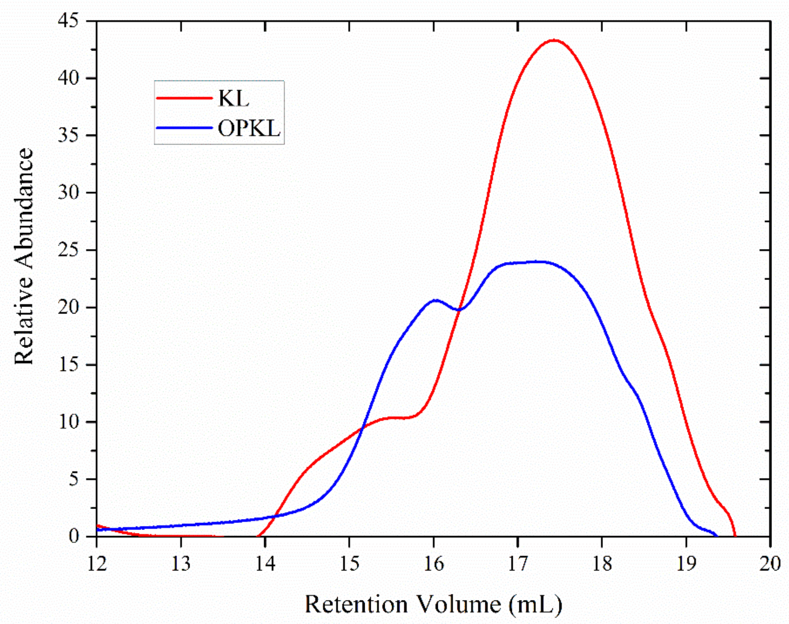

Figure S2. SEC chromatogram of kraft lignin and oxypropylated Kraft lignin

Kraft lignin showed the weight average molecular weight (Mw) of 5702 g mol^{-1} and number average molecular weight (Mn) of 1152 g mol^{-1} (Table S1). Oxypropylated lignin exhibited the Mw value of 4876 g mol^{-1} and Mn value of 876 g mol^{-1}, respectively. The polydispersity index of oxypropylated lignin was higher than kraft lignin. From the SEC chromatogram (Figure S2), it can be seen that that oxypropylated lignin is started to elute earlier than kraft lignin and it contains large portions of the high molecular weight fractions compared to the kraft lignin. The number average molecular weight of kraft lignin, Mn is higher than oxypropylated lignin which is also stressed that kraft lignin without modification contains large number of small molecular weight lignin fractions. After modification, polydispersity of oxypropylated lignin has been increased, explaining that oxypropylated lignin contains a broader molecular weight distribution range.

Table S1. The number average molecular weight (Mn), weight average molecular weight (Mw) and polydispersity index (Mw/Mn) of kraft lignin and oxypropylated kraft lignin

Sample	Mn (g mol^{-1})	Mw (g mol^{-1})	Mw/Mn
Kraft lignin	1152	5702	4.95
Oxypropylated kraft lignin	874	4876	5.58

Mechanical analysis of PLA and PLA-lignin composites

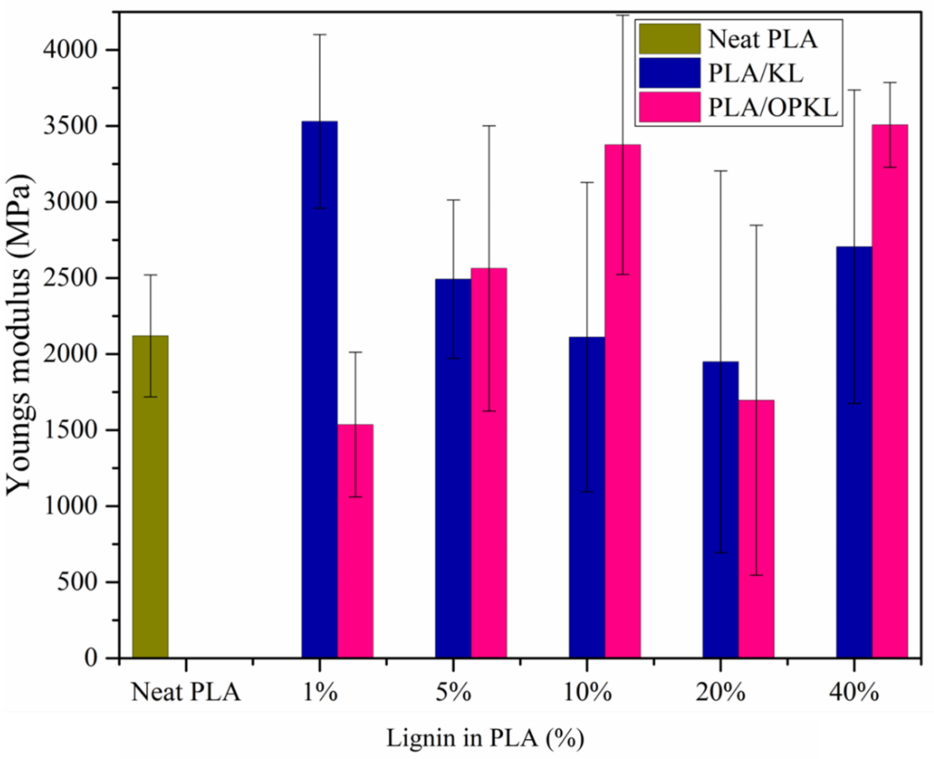

Figure S3. Young's moduli of neat PLA, PLA/KL and PLA/OPKL.

DSC analysis

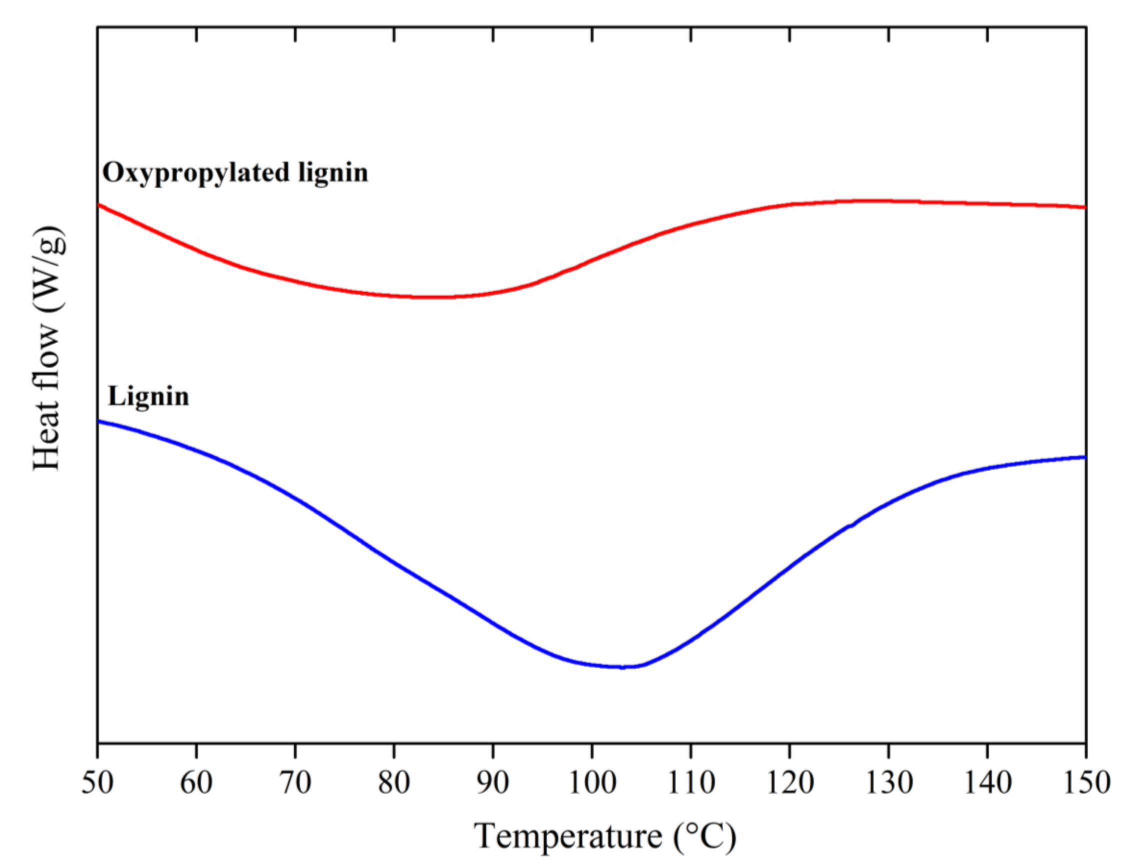

Figure S4: DSC curves of kraft lignin and oxypropylated kraft lignin
